# Numerical investigation of heat transfer in a garment convective cooling system

**DOI:** 10.1186/s40691-021-00276-3

**Published:** 2022-01-15

**Authors:** Yijie Zhang, Juhong Jia, Ziyi Guo

**Affiliations:** 1 Shangyu College, Shaoxing University, Shaoxing, 312300 Zhejiang China; 2 School of Fashion Design and Engineering, Zhejiang Sci-Tech University, Hangzhou, 310018 Zhejiang China; 3 School of Aerospace Engineering, Beijing Institute of Technology, Beijing, 100081 China

**Keywords:** Clothing microclimates, Human thermal comfort, Wearable cooling system, Convective heat transfer, CFD

## Abstract

A personal microclimate management system is designed to maintain thermal comfort which allows people to overcome a harsh environment. It consists of several micro-fans placed in the garment side seam to provide cooling air. The computational fluid dynamics method was used to simulate the three-dimensional model and analysis the influence of fan’s number and air gap distance. The obtained results depict that the introduced cool airflow will find its way along paths with flow resistance minimized and exhaust through several separated exit. The body heat flux is taken away at the same time. The convection effect is enhanced by the increase in the fans’ numbers, but the fans’ cooling effect varies a lot because of various air gap distances. When the air gap is small enough, the cooling air impact the body surface directly and causes fierce heat loss. While the air gap distance is large enough, the heat transfer along the skin surface could be enhanced by the eddy flow which is existed in the air gap between body and garment. These phenomena can maintain the body’s thermal comfort in a suitable range.

## Introduction

With the continuous spread of COVID-19, medical, community workers, and volunteers during their duties may be exposed to polluted air (Karim et al., 2020). They may also come into direct contact with patients and potential virus carriers. Therefore, special protective garments are widely used to block transmission. However, these tight, closed spaces between protective clothing and body surface make against human body heat dissipations and cause discomfort microclimate under clothing (Tang et al., 2016). The ordinary clothes are provided with holes for ventilation and heat dissipation, but this goal gains at the expense of losing security. A more acceptable way would be to design a personal microclimate management system to maintain thermal comfort which allows people to overcome a harsh environment (Cho et al., 2016). Such a system should maintain a comfortable microclimate, as well as filter and purify the inflow air. Moreover, the system should be user-controlled to adjust the ventilation rate in the air gap between the body and clothing.

The human body generates heat energy from the process of metabolism and body movements. While wearing ordinary work clothes, heat flux is lost easily from breathable cloth due to the high temperature gradient between the human body and ambient environment (Xu & Gonzalez, 2011). But under the closed protective clothing, the thermal energy is reserved in small air gap spaces because of airtight fabric. So, garment cooling measures must be considered to prevent thermal discomfort, excessive sweating even heat stroke results from the increase in human core body temperature.

Since clothing always remains close to the body even during dynamic motions, the portability of the air ventilation system is more advantageous. The portable garment cooling method can be classified into two categories: phase change garments and forced air exchanger garments. In phase change garments, the cooling effect result from the heat absorption by the melting of phase change materials (Salaun et al., 2010). Therefore, the cooling efficiency decreasing as the phase change material is melting. Furthermore, it is a difficult problem for the store and transmission of phase change materials, not to mention reusability. In a forced air exchange cooling system, some air exchange fans are installed on the garment surfaces, and heat loss occurs by convective heat transfer as well as evaporative moisture transfer (Delkumburewatte & Dias, 2012). Compared with the phase change system, the forced air exchange system has the advantage of relatively high cooling capacity and reusability. Besides, the simple manufacturing process and low cost are also its superiorities (Ernst & Garmella, 2013).

Previous studies showed that wearable air ventilation systems are effective enough till as high as 40 °C (Hadid et al., 2008) and 45 °C (Barwood et al., 2009) environmental temperatures. Some design modifications of air ventilation clothing by moving the fan locations and providing openings at appropriate locations were tried to improve the cooling performance (Zhao et al., 2013). The space between the skin and the clothing, i.e. the air gap, plays an important role in heat and mass transfer. Li et al. (2013) established a relationship between air gap thickness and heat transfer using 3D scanning and human body temperature data. Mert et al. (2015) studied the effect of heterogeneous and homogenous air gaps on dry heat loss through the garment by scanned cloud data. These experimental analyses, however, have some shortcomings because of the lack of three-dimensional distribution characters, which may bring about significant local variations. The computational fluid dynamics (CFD) method, with the ability to simulate 3D temperature and velocity fields, has become an essential tool in the prediction of thermal comfort of protective garments cooling systems (Zhang & Jia, 2021). Meanwhile, heat transfer and mass transfer can be acquired by solving the coupled multiple transfer model equations. Sun and Jasper (2015) investigated a 2D cooling system consists of a series of micro-fans, placed in a ribbon and attached to a garment. They argued that this convective cooling system significantly improved the convective and evaporative heat transfer coefficients when the inlet airflows were at 0.75 m/s and 1 m/s. Choudhary et al. (2019) developed a three-dimensional air ventilation cooling model to determine heat transfer between the human body and the environment by numerical simulation. The result showed that the area-weighted average torso heat flux increased while the fan airflow rate increased.

The above studies proved that the heat convection on body skin is highly affected by airflow conditions, such as air velocity, wind direction, turbulence eddies. To the best of our knowledge, few reports comprehensively describe the distribution and mechanism of airflow conditions between three-dimensional body skin and garment surface. In this research, a wearable convective cooling system composed of several micro-fans is proposed, then, the temperature and heat flux distributions, as well as flow mechanism are analyzed.

## Methods

### Model development

**Fig. 1:**
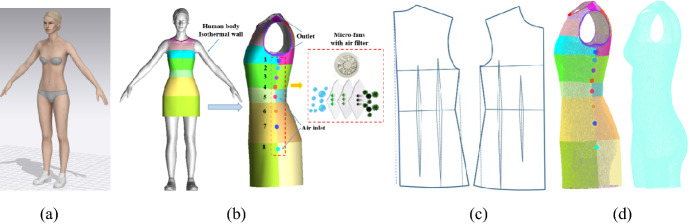
3D model and boundary conditions. **a** Geometry model, **b** garment type and boundaries, **c** garment pattern, **d** mesh domain

**Fig. 2:**
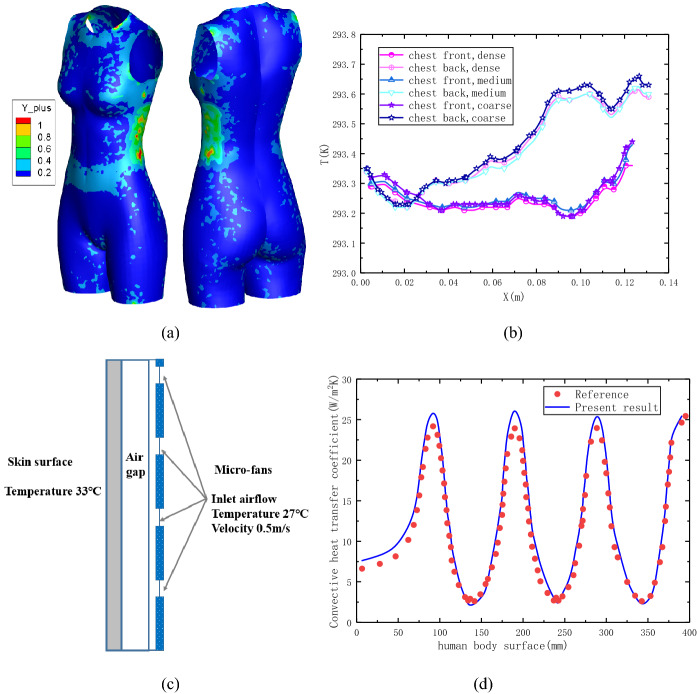
Mesh independence check and method evaluation. **a** The contours of *y*+ on body surface, **b** grid convergence tests, **c** wearable convective cooling system, **d** model validation with public results

A 3D avatar is used in the assessment of thermal comfort under different conditions. The female body is established by *CLO Standalone* (CLO Virtual Fashion Inc, Korea), with a height of 166 cm and bust, waist and hip girth length of 86, 66 and 90 cm, as shown in Fig. 1a. A fitted X-type garment is constructed by Bezier-spine curves. First, the body surface feature points are extracted and used to obtain corresponding garment feature points by adding air gap distance. Then, these garment feature points are used to construct garment surfaces by Bezier-spine curves (Zhang et al., 2014, 2018). The flatten garment pattern is shown in Fig. 2c. A wearable cooling system, consists of a series of filtered micro-fans with diameters of 2 cm, is embedded in the side seam of the protective garment. These fans connect to a potable lithium battery. This convective cooling system is expected to matain the thermal comfort in the hazardous environment. The Ansys ICEM-CFD (ANSYS Inc., Canonsburg, PA, USA) was used to generate an unstructured mesh in the computational domain by the robust Octree method. All the body and garment surfaces meshed with triangular surface mesh (Fig. 2d). Eight inflation layers were also generated along the body surface to capture the near wall temperature gradient correctly. It is to be noted here that only half-geometry was taken into consideration in this study due to the symmetric, and treated as the computational domain.

### Governing equations

A computational fluid dynamics approach was used to perform three dimensional transient simulations of flow and heat transfer through the microclimate involving air ventilation system. Some of the important simplifications and assumptions considered in the present study are as follows (Ismail et al., 2019; Oh & Kato, 2018): Firstly, we assume that the present model deals with dry conditions without sweating, i.e. we focus on convective heat transfer rather than evaporative heat transfer. Secondly, the protective garment was considered air-tight, the air flows in from the micro-fans and goes out through collar, sleeves and hem. Thirdly, we also assume turbulent incompressible flow with ideal gas, as well as the no-slip boundary condition for the wall surface. The Boussinesq approximation was used to calculate the buoyancy effect, and realizable *k–ɛ* model was used to deal with the turbulence effect.

With these assumptions, the governing Reynolds-averaged equations are summarized below (Renato et al., 2019):

Continuity equation:

1 $$ \frac{\partial }{{\partial x_{j} }}(\rho u_{j} ) = 0 $$

Momentum equation:

2 $$ \frac{{\partial (\rho u_{i} u_{j} )}}{{\partial x_{j} }} = - \frac{\partial p}{{\partial x_{i} }} + \frac{\partial }{{\partial x_{j} }}\left[ {\mu \left( {\frac{{\partial u_{i} }}{{\partial x_{j} }} + \frac{{\partial u_{j} }}{{\partial x_{i} }}} \right) - \rho \overline{{u_{{_{i} }}^{*} u_{{_{j} }}^{*} }} } \right]{ + }\rho \beta \left( {T - T_{0} } \right)g_{i} $$

Energy equation:

$$ \frac{{\partial (\rho u_{i} c_{p} T)}}{{\partial x_{i} }} = - \frac{\partial }{{\partial x_{i} }}\left( {k\frac{\partial T}{{\partial x_{i} }} + \rho \overline{{u_{i} }} c_{p} \overline{T} } \right) $$

The *k–ɛ* turbulence model is described by the turbulent kinetic energy *k* and its dissipation rate *ɛ*, which can be expressed as follows (Yang et al., 2016):

4 $$ \frac{\partial }{{\partial x_{i} }}\left( {\rho ku_{i} } \right) = \frac{\partial }{{\partial x_{{\text{j}}} }}\left[ {\left( {\mu + \frac{{\mu_{t} }}{{\sigma_{k} }}} \right)\frac{\partial k}{{\partial x_{j} }}} \right]{ + }\frac{{\mu_{t} }}{2}\left( {\frac{{\partial u_{i} }}{{\partial x_{j} }} + \frac{{\partial u_{j} }}{{\partial x_{i} }}} \right) - \rho \varepsilon $$

5 $$ \frac{\partial }{{\partial x_{i} }}\left( {\rho \varepsilon u_{i} } \right) = \frac{\partial }{{\partial x_{j} }}\left[ {\left( {\mu + \frac{{\mu_{t} }}{{\sigma_{\varepsilon } }}} \right)\frac{\partial \varepsilon }{{\partial x_{j} }}} \right] + C_{1\varepsilon } \frac{\varepsilon }{k}\frac{{\mu_{t} }}{2}\left( {\frac{{\partial u_{i} }}{{\partial x_{j} }} + \frac{{\partial u_{j} }}{{\partial x_{i} }}} \right) - C_{2\varepsilon } \rho \frac{{\varepsilon^{2} }}{k} $$

where, the viscosities turbulence coefficient $\mu_{t}$ is defined as

$$ \mu_{t} = \rho C_{\mu } \frac{{k^{2} }}{\varepsilon },\;{\text{and}}\quad C_{\mu } = 0.09,C_{1\varepsilon } = 1.44,C_{{{2}\varepsilon }} = 1.92, \, \sigma_{k} = 1, \, \sigma_{\varepsilon } = 1.3. $$

### Boundary conditions and technical approaches

The boundary conditions are shown in Fig. 1b. The micro-fans are considered as airflow inlet, and the air gaps of armhole and neckline are considered as air outflow. The human body is set as isothermal wall, the body skin surface temperature was fixed at 36 °C/309 K. At the inlet, so as to investigate the heat transfer in the microclimate of protective clothing, forced convection in a normal atmospheric environment, ambient temperature of 20 ℃/293 K, was modeled by considering air flowing through micro-fans then reach the body surface. The velocity was kept constant at 0.5 m/s, and the relative humidity was maintained at 40%. The following calculations were performed under dry conditions (i.e. no sweating). A no-slip condition at the surface of the garment and body skin was imposed. The thermal conductivity of air and body are considered as 0.026 and 0.3 W/(m K). The convective heat transfer from the outside of the garment to the environment was also considered and treated as a typical natural convection condition. This heat loss coefficient was taken as 5 W/m^2^ K. The textile consisted of a 100% cotton fabric layer of thickness 0.204 mm, and the physical properties were obtained from published results (Sun & Jasper, 2015). The detailed physical properties and boundary conditions are shown in Table 1.

**Table 1:** Physical properties and boundary conditions

	Medium	Boundary types	Physical properties	Thermal conductivity (W/K m)	Pressure constant thermal capacity (J/K kg)
Inlet	Air	Uniform inlet flow	p_air_ = 101325 pa T_air_ = 283 K v_air_ = 0.5 m/s ρ_air_ = 1.165 kg/m^3^	0.026	1000
Outlet	Air	Back pressure outlet	p_out_ = 101325 pa	–	–
Body surface	Human skin	Isothermal wall	T_skin_ = 310 K ρ_skin_ = 860 kg/m^3^	0.3	5021
Garment	Cotton	Convection wall	ρ_cottom_ = 81 kg/m^3^	0.059	1150

The above mentioned mathematical equations were solved at each node of the computational domain by using the finite volume method (FVM). A second order upwind scheme for all the pressure, momentum, turbulence and energy equations were used for discretization with a multidimensional total variation diminishing flux limiter (Jia et al., 2020). Finite volume method based commercial CFD solver CFD++ (Metacomp Technologies Inc, USA) was used to obtain the numerical results. Double precision format was used for all kinds of computations and the converged residual level was set to below 1 × 10^–5^.

### Methodologies evaluation

The nondimensional near wall distance *y*+, which express as $y^{ + } { = }\rho u_{\tau } y_{wall} /\mu$, was used to measure the first grid cell above the wall. As can see in Fig. 2a, the near wall *y*+ was less than 1.0 for all most of the human body surface. At this time, the first grid cell was located within the viscous sublayer (Jia et al., 2020). Grid independence on cooling performance of micro-fan system was analyzed with three levels of mesh refinement, namely 3.2 million (coarse), 3.7 million (medium) and 4.2 million nodes (dense). The results were shown in Fig. 2b, it can be observed from the mesh check study that the dense grid is enough to resolve the heat transfer of the micro-fan cooling system.

The numerical method is validated first against the available results (Sun & Jasper, 2015) for a two dimensional wearable convective cooling system, in which a series of micro-fans installed in a ribbon and attached to clothing, as shown in Fig. 2c. The convective heat transfer coefficient was obtained and compared with public results. The comparison study shows that the present work is agrees with the available results well. So, it would be satisfactory to carry out the following calculations under the above conditions.

## Results and Discussion

**Fig. 3:**
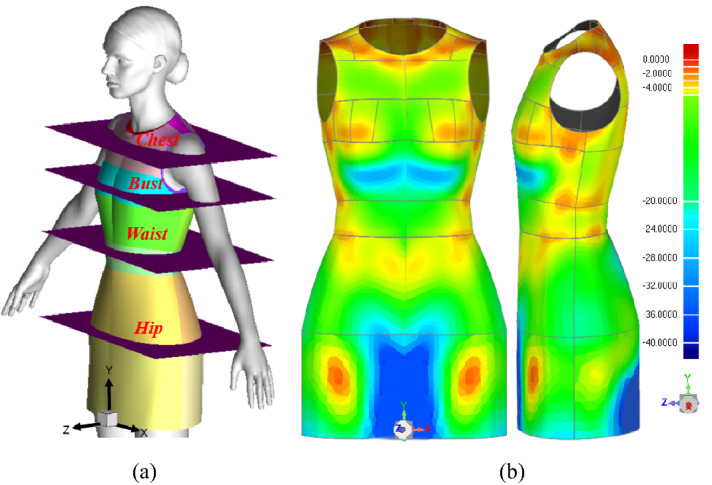
Landmarks and air gap. **a** Horizontal cross-section and cut lines, **b** air gap distance contours

With the aim of analyzing the temperature and heat transfer distribution in detail, four parallel cross-sections were created, namely the chest, bust, waist and hip horizontal planes. Then, the key girths were generated as the intersections of plane and body surface, as shown in Fig. 3a. The Cartesian coordinates are fixed to the symmetry plane, and the X, Y, Z refers to the horizontal, vertical and radial distance.

Three different types of calculations were carried out to study the heat exchange between the body and clothing and to determine heat flux quantitatively along the skin surface, as: (1) one was comprised of double 2 cm diameter fans, namely the fans of No. 3 and No. 7, located in the lower bust and waist with a distance of 18 cm; (2) the second was comprised of four 2 cm fans, i.e., the fans of No. 1, No. 3, No. 5 and No. 7, located in the lower armpit, bust, waist and hip with the distance of 8, 9, and 14 cm and (3) the third was comprised of eight 2 cm fans (No. 1 to No. 8) located in lower armpit, bust, waist and hip with the distance of 3.5, triple 4.5, 5.5, 6.5, 7.5 and 10 cm on the garment surface. The fan’s type and numbers are shown in Table 2. The ambient air temperature is 283 K and the inflow air is transfer through micro-fans with a constant velocity of 0.5 m/s.

**Table 2:** Calculation conditions

Cases	Fan type (cm)	Inlet airflow (m/s)	Micro-fans	Numbers	Space between fans
Case 1	2	0.5	2	No. 3, No. 7	18 cm
Case 2	2	0.5	4	No. 1, No. 3, No. 5 and No. 7	8, 9, and 14 cm
Case 3	2	0.5	8	No. 1 to No. 8	3.5 cm, triple 4.5 cm, 5.5 cm, 6.5 cm, 7.5 cm and 10 cm

### Three dimensional air gap distributions

**Fig. 4:**
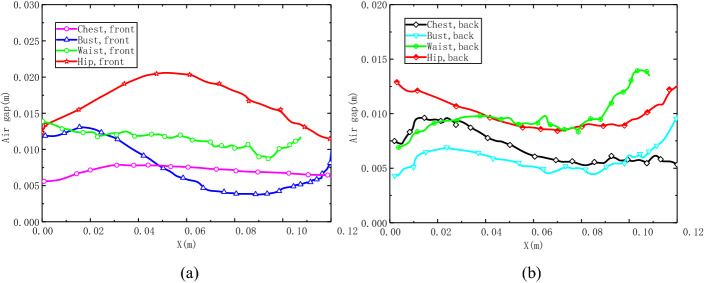
Air gap distance distributions. **a** Air gap distance of front side, **b** air gap distance of back side

The 3D air gap contours are shown in Fig. 3b. As shown, the neckline, side bust and waist yield a narrow air gap of less than 5 mm because the clothing is closed to the body surface. Meanwhile, the underbust and groin region have a large air gap distance due to the structure of the human body. The detailed air gap distributions on body key girth are shown in Fig. 4. As can see in Fig. 4a, the curves of the chest and bust girth go down along the body surface on the front side. However, the trend of the hip front girth curve is increasing at first and then decreasing. The reason is that this region is nearby the area of the human body groin. Figure 4b shows the scene of body backside, the air gap is keep decreasing on the whole, but the distance is bigger than that on the front side.

### Temperature distributions on garment surface

**Fig. 5:**
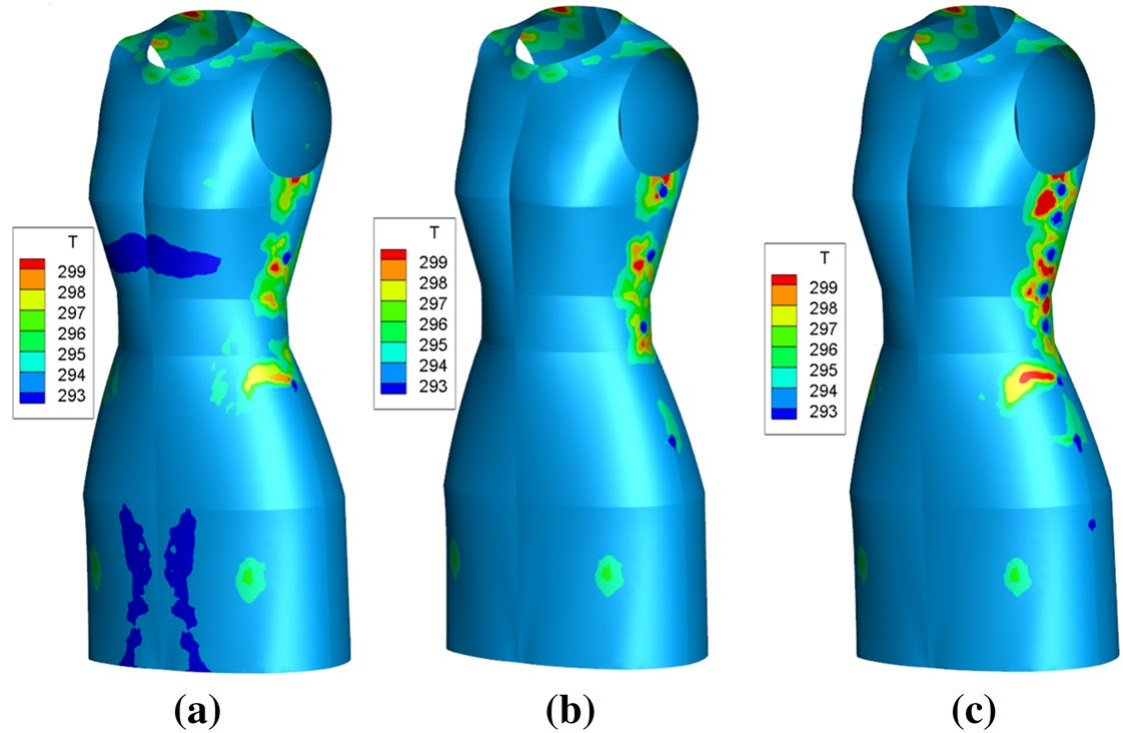
Temperature contours under various micro-fans type. **a** Case 1, 2 fans, **b** case 2, 4 fans, **c** case 3, 8 fans

The simulated temperature contours of different micro-fans configurations are shown in Fig. 5. As can see, there are several high temperature regions on the clothing surface near the neckline, armpit, waist and thigh under the natural convection condition. The reason is that the above mentioned area is narrow enough so that the heat flux transfer from the body to the clothing surface easily. But Fig. 5a–c show different scene because of forced heat flux convection by micro-fans. Firstly, if there are just two separated micro-fans installed on the garment which are located at the lower bust and upper thigh (Fig. 5b), there are other two high temperature areas appear around the micro-fans. Meanwhile, there is a big high temperature region on the thigh girth near the fan, the reason is mainly that part of the inlet air flows reflected the garment surface due to the narrow air gap distance, as can see in Fig. 3b. Secondly, if there are four micro-fans located at the armpit, underbust, waist and upper hip (Fig. 5c), the high temperature regions become bigger and the peak value increase to a new level and the area near the fourth fan keeps at a low level because of a big air gap distance. Thirdly, if the number of micro-fans increased to eight, there are several anomaly high temperature regions around No. 1 to No. 6 fans because of the narrow and uneven air gap. However, the high temperature regions are not so apparently around the last two fans because of wide air gap.

**Fig. 6:**
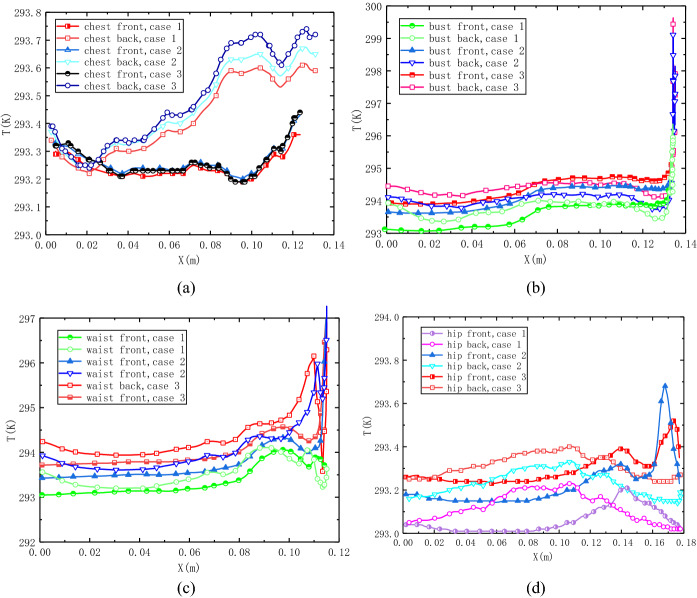
Temperature distribution on garment surface. **a** Chest girth, **b** bust girth, **c** waist girth, **d** hip girth

In order to analyze the temperature quantitatively, the detailed temperature distributions on key girths are exhibited in Fig. 6. As is shown in Fig. 6a, the temperature distributions on the chest girth garment surface increase with the horizontal distance nonlinear. The case of two fans yield the lowest temperature on the both side of garment surface, while the model with eight fans obtains the highest temperature, especially on the back side. Figure 6b shows that, the temperature curves on the bust girth of three cases keep pace with each other on the whole, except at the location of armpit. On the armpit area, the temperature curves rise up quickly and reach about 299.5 K for case 3, 298.6 K for case 2 and 296.8 K for case 1. This phenomenon mainly due to the combined action of narrow distance and quickly flow speed of outflow. Figure 6c depicts that the temperature curves show a wavelike appearance on the waist girth, especially between the section of 0.1 m < *x* < 0.12 m. To be specific, there is a plateau region on the temperature curve of case 1, but there are two high peaks for case 2 and 3 where the temperature reach to 295.9 K and 296.8 K. As can see in Fig. 6d, there show different scene for front side and back side. The temperature curves of back hip side increase to high values in the middle but decrease to lower values at the end. However, the temperature curves of front hip side decrease to a low level at the middle due to large air gap distance, but increase to high number near the side part because of forced convection of micro-fans. Moreover, the convection effect is enhanced by the increase the number of fans.

### Heat transfer on body surface

**Fig. 7:**
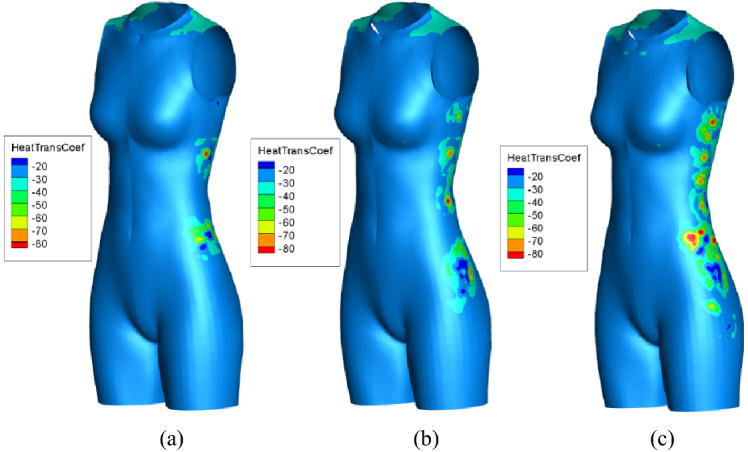
Heat flux contours for different cases. **a** Case 1, two fans, **b** case 2, four fans, **c** case 3, eight fans

The conductive and convective heat transfer coefficients for an environmental temperature of 20 °C according to simulation are given in Fig. 7. Predictably, the heat transfer distribution on the body surface is strongly related to the inflow air from micro-fans. Only a small portion of body surface is covered by micro-fan, but a large high heat flux area is around it due to three dimensional airflow. As can see in Fig. 7a, under the circumstance of case 1, because of the forced convective heat transfer, there are two high heat flux regions around the micro-fans, and each of them has a peak value of about 85 W/m^2^ and 60 W/m^2^. Besides, there is also a high heat flux region on the top of the shoulder, mainly due to the conductive in air gap. For case 2 in Fig. 7b, there are four different high heat flux regions around the micro-fan holes, and each of them has a heat spot corresponding to the micro-fans inlet air flow. As for case 3 in Fig. 7c, eight high heat flux regions next to each other along the side seam. It is interesting to note that the high heat spots area show a scattered appearance, and the location of the high heat spot is offset from the original position on the waist and thigh part of the body. This phenomenon will be further explored in detail in followed chapter.

**Fig. 8:**
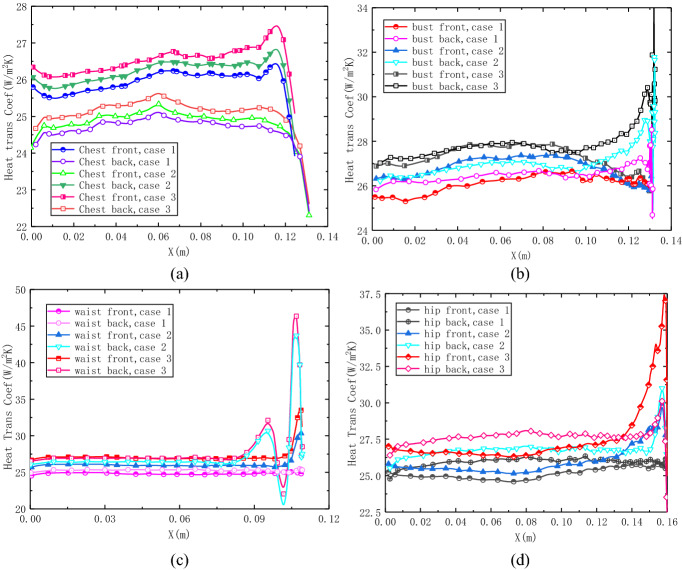
Heat transfer coefficient distribution on body surface. **a** Chest girth, **b** bust girth, **c** waist girth, **d** hip girth

In order to investigate the heat transfer performance quantitatively, the heat transfer coefficients on key girths are shown in Fig. 8. The average heat transfer on chest girth, in Fig. 8a, is about 27 W/m^2^ K on the back side and 28.5 W/m^2^ K on the front side. The heat flux curves have a plateau on the front side and a peak on the back side for all the three types of micro-fans, and the heat transfer coefficients are higher for more fans, as case 3 yield the highest peak value of 29.4 W/m^2^ K. Figure 8b depicts that the case of more fans model gains a bigger heat flux on the bust girth, especially on the position near the fans. Figure 8c indicates that the variety of fans number has less influence on the waist girth of 0 m < *x* < 0.75 m, for the heat transfer coefficient almost keeps the same for different models. But the area near the micro-fan inlet flow has a totally different scene, the curves have sinusoid shape for four and eight fan models. The fans locations correspond to the peak values of convective heat transfer coefficient in these regions. Figure 8d shows that the heat flux keep at a low level of about 26 W/m^2^ K for the front side and 27 W/m^2^ K for the back side on most of the hip area. But due to the forced convection, the area near the fans yields a peak value of more than 27 W/m^2^ K for the eight fans model. From the above analysis, we find that the micro-fan brings fierce convection and large heat loss around the fan holes. The heat transfer strength is highly related to the fan numbers. However, the influence of forced convection is decreased while the location keeps away from the fans mainly due to the low efficiency air ventilation in the complex air gap.

### Flow mechanism analysis

**Fig. 9:**
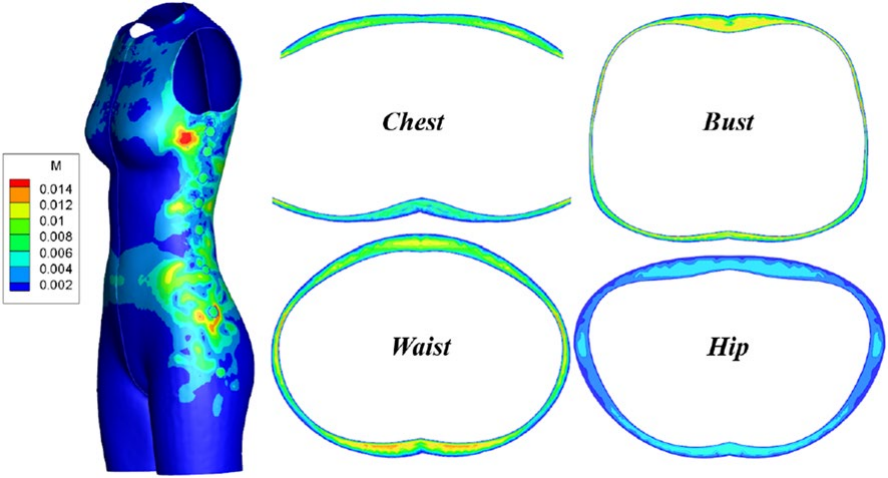
Air flow contours of air gap and body surface

To illustrate the airflow properties and eddies formed near the skin surface. The local velocity profile is obtained and shown in Fig. 9. As can see, the cooling air from micro-fan gives rise to the high speed airflow in the area around the fans. These flows carry the heat flux from body surface and reach the garment surface. This action will bring about the temperature rise on garment surface. However, the air velocity goes down quickly while the location moves far away from the fans. So, the air ventilation efficiency in these regions is very poor, as has been predicted above.

**Fig. 10:**
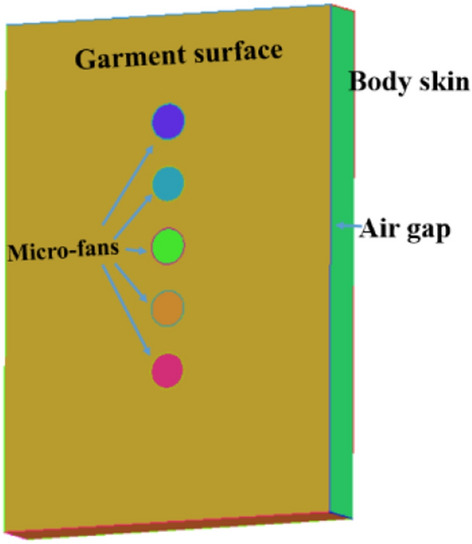
Parallel flats model

With the aim of investigating the detailed flow mechanism, the airflow between two parallel flats is computed to simulate the body and garment surfaces with the air gap distance of 30 mm and 5 mm, as shown in Fig. 10. There are several micro-fans installed on the first flat with a diameter of 2 cm. The boundary conditions are set to be the same as the human body model.

**Fig. 11:**
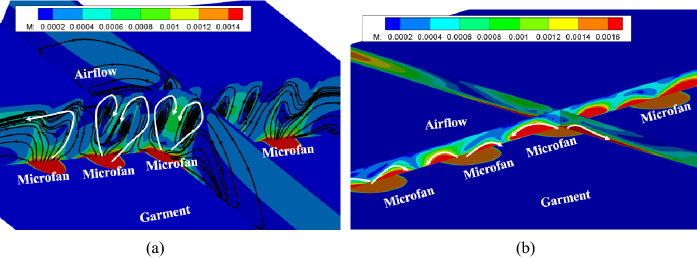
Regional air flow contours under different air gap distance. **a** Air flow contours with 30 mm air gap, **b** air flow contours with 5 mm air gap

The calculated airflow speed contours are shown in Fig. 11. As can see in Fig. 11a, when the air gap distance is large enough, i.e., 30 mm, the cooling air will flow through the fan and reach the skin surface, then two eddy vertexes are created in the air gap. The air speed in eddies is relatively slow, so it would cause a small part of heat transfer loss on body surface. However, when the air gap distance is small enough, namely 5 mm, as shown in Fig. 11b, the inflow will impact the body surface, then it is separated into several sub-flows in different directions. These sub-flows from different fans may interact with each other and cause several high speed areas on the body surface. Meanwhile, this fierce impact brings about high heat loss on the body surface. This phenomenon can maintain a lower body surface temperature, but fails to maintain a comfortable micro-environment.

## Conclusions

With the aim of keeping thermal comfort in a harsh environment, a concept of a lightweight wearable convective cooling system is investigated. This system consists of a series of filtered micro-fans which is embedded in side seam of the protective garment. Three different configurations, namely two, four or eight 2 cm micro-fans models, were analyzed. Three dimensional heat transfer at the body surface was simulated with the inflow air speed of 0.5 m/s. The main conclusions are as follows:

The installment of micro-fans is a feasible way that can maintain a comfortable microclimate under protective garments. The air flow finds its way along paths with flow resistance minimized and takes away the body heat flux.

The micro-fan brings fierce convection and large heat loss around the fan holes and the convection effect is enhanced by the increase of the fans numbers. However, the influence of forced convection is decreased while keeps away from the fans hole due to the low efficiency air ventilation in the complex air gap.

When the air gap is small enough, the cooling air impact the body surface directly and cause fierce heat loss. At this time, the thermal comfort would goes down to some extent. When the air gap distance is large enough, the heat transfer along the skin surface could be enhanced by the eddy flow which is existed in the air gap between body and garment. Under this circumstance, thermal comfort can be improved to a large extent.

## Data Availability

Not applicable.
